# Long working hours and psychiatric treatment: A Danish follow-up study

**DOI:** 10.5271/sjweh.3936

**Published:** 2021-03-31

**Authors:** Harald Hannerz FilLic, Karen Albertsen, Martin Lindhardt Nielsen, Anne Helene Garde

**Affiliations:** National Research Centre for the Working Environment, Copenhagen, Denmark; TeamArbejdsliv ApS, Valby, Denmark; Lægekonsulenten.dk, Viby J, Denmark; Department of Public Health, University of Copenhagen, DK-1014 Copenhagen, Denmark

**Keywords:** anxiety, mood disorder, occupational health, prescription drug, psychiatric hospital treatment, psychotropic medicine, stress-related disorder

## Abstract

**Objective:**

This study aimed to estimate prospective associations between long working hours and (i) redeemed prescriptions for psychotropic drugs and (ii) psychiatric hospital treatment due to mood, anxiety or stress-related disease, among full-time employees in Denmark.

**Methods:**

Full-time employees who participated in the Danish Labor Force Survey sometime in the period 2000–2013 (N=131 321] were followed for up to five years in national registers for redeemed prescriptions for psychotropic drugs and psychiatric hospital treatment due to mood, anxiety or stress-related disease. Rate ratios (RR) were estimated for 41–48 versus 32–40 and >48 versus 32–40 working hours a week. The analyses were controlled for sex, age, night shift work, calendar time of the interview and socioeconomic status (SES). Prevalent cases were excluded in primary analyses.

**Results:**

The RR for psychotropic drugs were estimated at 0.94 [99% confidence interval (CI) 0.88–1.01] for 41–48 versus 32–40 working hours a week and 1.08 (99% CI 0.99–1.18) for >48 versus 32–40 working hours a week. The corresponding RR for psychiatric hospital treatments were estimated at 0.90 (95% CI 0.75–1.08) and 0.96 (95% CI 0.76–1.21). We did not find any statistically significant interaction between weekly working hours and age, sex, SES or night shift work.

**Conclusion:**

Long working hours as they occur in in the general working population of Denmark are not an important predictor of mental ill health.

The governments of Japan, Taiwan and South Korea recognize extra-long working hours as a compensable risk factor for mental disorders (1). In a meta-analysis by Virtanen et al (2), the relative risk of developing depressive symptoms among workers with long hours (most often defined as ≥55 weekly hours) versus standard hours (most often 35–40 weekly hours) in Asian countries was estimated at 1.50 [95% confidence interval (CI) 1.13–2.01]. In a recent questionnaire-based study of employees at training hospitals in Japan, the relative risks of having developed depressive symptoms at a three-months follow-up were estimated at 2.83 for 80–99.9 versus ≤60 working hours a week and at 6.96 for ≥100 versus ≤60 working hours a week (3).

In western countries, long working hours are most often not as excessive as in some Asian countries and do not appear to be a similarly serious public mental health problem. In the aforementioned meta-analysis by Virtanen et al (2), the risk ratios (RR) for development of depressive symptoms between workers with long versus standard working hours were estimated to be 1.11 (95% CI 1.00–1.22) in Europe, 0.97 (95% CI 0.70–1.34) in North America, and 0.95 (95% CI 0.70–1.29) in Australia.

In a previous study, we used RR of redeemed prescription for psychotropic drugs to estimate prospective associations between long working hours and mental ill-health among employees in the general working population in Denmark during 99 018 person-years at risk (4). RR for the contrasts 41–48 versus 32–40 and >48 versus 32–40 working hours a week were estimated with and without stratification by age, gender, socioeconomic status (SES) and shift work, respectively. None of the results was statistically significant after adjustment for multiple comparisons. The study suggested, however, that overtime work that exceeds the limit of the EU working time directive (>48 working hours a week) (5) might be associated with a slightly increased risk among employees in the general population (RR 1.15, 95% CI 1.02–1.30). It suggested, moreover, that >48 working hours a week may be an important risk factor among shift workers (RR 1.51, 95% CI 1.15–1.98).

The aim of the present study was firstly to replicate the estimations of our previous study in a larger data material and secondly to supplement the examination with estimated RR for psychiatric hospital treatment due to mood, anxiety or stress-related disease.

## Methods

In the present paper, we will only give a brief description of the material and methods of the study. A detailed description can be found in our study protocol (6), which was peer-reviewed and published before we commenced with the analysis. The protocol defines two major studies, one of them focuses on effects of long working hours (reported here) while the other focuses on effects of night shift work (results to be reported elsewhere).

### The data material

Individual participant data on usual weekly working hours and night shift work were extracted from the Danish Labor Force Survey (DLFS). The DLFS data were thereafter linked to person-based data on redeemed prescriptions, psychiatric hospital treatments, industry, socioeconomic status, migrations and deaths, from national registers, which cover the entire population of Denmark. The DLFS is based on random samples of 15–74 years old inhabitants of Denmark that have been drawn each quarter of each calendar year since 1994. During the time-period spanned by the present study, the data were collected by means of telephone interviews. The selected participants were invited to be interviewed four times during a period of approximately 1.5 years (7). The response rates have decreased with time, from 70% in 2002 to 53% in 2013. The primary analyses of the present study are based on the participants’ first interview in the calendar period 2000–2013.

### Inclusion criteria

The study included people who (i) responded to DLFS sometime during the calendar years 2000–2013; (ii) were 20–59 years old at the start of the follow-up period; (iii) were employed with 32–100 usual working hours a week at the time of the interview; and (iv) did not receive psychiatric hospital treatment for any type of mental disorder (ICD-10: F00-F99), as principal diagnosis and did not redeem a prescription for any type of psychotropic drug (ATC: N05-N06) during a one-year period preceding the start of the follow-up period.

### Clinical endpoints

The following endpoints were regarded: (i) redeemed prescriptions for any type of psychotropic medicine, ie, drugs in the ATC-code category N05 (psycholeptica) or N06 (psychoanaleptica); and (ii) psychiatric hospital treatment with a mood, anxiety or stress-related disorder (ICD-10: F30 – F41 or F43) as principal diagnosis.

### Weekly working hours

The participants of DLFS were asked first how many hours a week they usually work in their primary job and then (if relevant) how many hours they usually work in each of their secondary jobs. In the present study, we added the hours worked in the primary and secondary jobs in order to form the exposure variable “weekly working hours”. Further details are given in our study protocol (6).

### Follow-up

The follow-up started at the end of the calendar year of the participant baseline interview and ended whenever any of the following events occurred: (i) five years had elapsed since start of follow-up; (ii) the subject reached the clinical endpoint of the analysis; (iii) the subject emigrated; (iv) the subject died; (iv) the study period ended (31 December 2014 for psychotropic medicine; 31 December 2017 for psychiatric hospital treatment).

### Statistical analyses

Poisson regression was used to estimate rates of redeemed prescriptions for psychotropic medicine and psychiatric hospital treatment due to mood, anxiety or stress-related disorders, separately, as a function of weekly working hours [32–40 (reference); 41–48; >48 hours/week]. The analyses were controlled for night shift work (yes versus no), sex, age (10-year classes), calendar time of the interview (2000–2004; 2005–2009; 2010–2013) and SES (legislators, senior officials and managers; professionals; technicians and associate professionals; workers in occupations that require skills at a basic level; workers in elementary occupations; and gainfully occupied people with an unknown occupation). The logarithm of person years at risk was used as offset. Likelihood ratios were used to test for statistical significance.

For redeemed prescriptions of psychotropic medicine, we tested the following effects, each at the significance level 0.01: (i) main effect of weekly working hours; (ii) effect of interaction between age and weekly working hours; (iii) effect of interaction between sex and weekly working hours; (iv) effect of interaction between SES and weekly working hours; and (v) effect of interaction between night shift work and weekly working hours.

For psychiatric hospital treatment due to mood, anxiety or stress-related disorders, we tested for a main effect of weekly working hours at the significance level 0.05. We did not have the statistical power necessary to test for interaction effects.

RR for the main effects of weekly working hours were estimated for redeemed prescriptions of psychotropic drugs as well as for psychiatric hospital treatments. The RR for redeemed prescriptions were, moreover, firstly stratified by sex, age, night shift work and SES and thereafter pooled with the corresponding results from our previous study on associations between weekly working hours and redeemed prescriptions for psychotropic drugs in the general population of Denmark 1995–2010 (4).

The pooling procedure is described in our study protocol (6) as follows: “The pooled results will be obtained through inverse-variance weighting. Since the present study has the same target population as our previous study and the study periods are overlapping, it is likely that some of the participants in our previous study also have participated in the DLFS. Based on the number of participants in our previous study in relation to the number of people in the target population, we expect that approximately one percent of the participants of the present study also participated in our previous study. This overlap will be taken into account in the pooling of the results by use of the following strategy: Before the results are pooled, the standard error of the present study will be multiplied by the square root of (1/(1-x)), where x=0.01 is the proportion of the participants in the present study that are likely to have participated in our previous study.”

Sensitivity analyses were conducted to: (i) compare results obtained with and without exclusion of former and current cases of psychiatric treatment, (ii) compare results obtained with and without control for industrial sector, and (iii) find out if the estimated strength of the association between weekly working hours and redeemed prescriptions for psychotropic drugs would increase when exposure is more stable over time. The methods and results of the sensitivity analyses are given in the supplementary material (www.sjweh.fi/show_abstract.php?abstract_id=3936).

In the sensitivity analyses, we estimate a series of RR and present these with 99% CI. Here, it should be noted that we do not regard the sensitivity analyses and their CI as statistical significance tests. The results of the sensitivity analyses will therefore never be regarded as statistically significant. They may, however, strengthen, weaken or invalidate statistical conclusions of the primary analyses.

## Results

A total of 341 482 persons participated in the DLFS sometime during the time period 2000–2013. Of these, we excluded: 99 736 for not being 20–59 years old, 63 914 for not being employed, 33 571 for working <32 hours per week, 292 for reporting to work >100 hours per week, 436 for emigration or death during the calendar year preceding start of follow-up, 10 for not being found in national registers, 668 for missing data on night-time work, and 11 534 for redeemed prescriptions for psychotropic drugs or use of psychiatric hospital treatment during the calendar year preceding start of follow-up. The remaining 131 321 participants were included in the primary analysis. Among the included, we observed a total of 15 826 cases of redeemed prescriptions for psychotropic drugs in 521 976 person years at risk and 1480 cases of psychiatric hospital treatment due to mood, anxiety or stress-related disease in 636 673 person years at risk. Of the hospital treatment cases, 22% were inpatients, 53% were outpatients and 25% were emergency ward patients. The diagnoses among the cases were distributed as follows: F30 manic episode, 0.5%; F31 bipolar affective disorder, 2.4%; F32 depressive episode, 25.8%; F33 recurrent depressive disorder, 10.2%; F34, F38, F39 persistent, other or unspecified affective mood disorders, 0.5%; F40 phobic anxiety disorders, 3.0%; F41other anxiety disorders, 8.6%; F43 reaction to severe stress, and adjustment disorders, 48.9%.

We found a statistically significant relationship between weekly working hours and redeemed prescriptions for psychotropic drugs (P=0.0031), with RR estimated at 0.94 (99% CI 0.88–1.01) for 41–48 versus 32–40 hours a week and 1.08 (99% CI 0.99–1.18) for >48 versus 32–40 hours a week. Here it should be noted that the P-value rejects the null hypothesis of no association between weekly working hours and redeemed prescriptions for psychotropic drugs. In other words, it rejects the null hypothesis, which states that there are no rate differences among the three working hour categories. The statistical significance is due to the difference between the categories >48 versus 41–48 working hours a week (RR 1.15, 99% CI 1.03–1.28).

The association between weekly working hours and psychiatric hospital treatment due to mood, anxiety or stress-related disease was statistically non-significant (P=0.52), with RR estimated at 0.90 (95% CI 0.75–1.08) for 41–48 versus 32–40 hours a week and 0.96 (95% CI 0.76–1.21) for >48 versus 32–40 hours a week.

We tested interaction effects on redeemed prescriptions for psychotropic drugs but not on psychiatric hospital treatment. We did not find any statistically significant interaction between weekly working hours and age (P=0.86), sex (P=0.56), SES (P=0.24), or night shift work (P=0.26).

The result of the present primary analysis of redeemed prescriptions for psychotropic drugs as well as results obtained after pooling with our previous study (4) are shown in table 1. The result of the analysis of psychiatric hospital treatment are shown in table 2.

**Table 1:** Rate ratio (RR) with 99% confidence interval (CI) for incident use of psychotropic drugs, as a function of weekly working hours among employees in Denmark in the calendar years 2000–2013.

Type of population (weekly working hours)	The present study				Hannerz & Albertsen (2016)		Pooled results	
		
	Person years	Cases	RR	99% CI	RR	99% CI	RR	99% CI
All workers ^a^								
>48	32 718	978	1.08	0.99–1.18	1.15	0.98–1.35	1.09	1.01–1.18
41–48	57 164	1568	0.94	0.88–1.01	1.04	0.92–1.19	0.96	0.90–1.02
32–40	432 094	13 280	1.00		1.00		1.00	
Male workers ^b^								
>48	25 873	686	1.06	0.95–1.17	1.13	0.92–1.38	1.07	0.98–1.18
41–48	36 306	811	0.92	0.83–1.01	0.97	0.80–1.18	0.93	0.85–1.02
32–40	223 086	5466	1.00		1.00		1.00	
Female workers ^b^								
>48	6845	292	1.12	0.96–1.31	1.16	0.87–1.53	1.13	0.99–1.30
41–48	20 858	757	0.96	0.87–1.06	1.11	0.93–1.33	0.99	0.91–1.08
32–40	209 008	7814	1.00		1.00		1.00	
Workers with night/shift work ^c^								
>48	9514	290	1.04	0.88–1.23	1.51	1.06–2.16	1.12	0.96–1.30
41–48	8211	247	1.03	0.86–1.23	1.18	0.81–1.71	1.05	0.90–1.24
32–40	47 833	1489	1.00		1.00		1.00	
Workers without night/shift work ^c^								
>48	23 204	688	1.10	0.99–1.21	1.08	0.90–1.30	1.09	1.00–1.19
41–48	48 953	1321	0.92	0.86–1.00	1.03	0.89–1.18	0.95	0.89–1.01
32–40	384 261	11 791	1.00		1.00		1.00	
Legislators, senior officials, and managers ^d^								
>48	3732	97	1.02	0.76–1.39	1.31	0.61–2.83	1.06	0.80–1.41
41–48	3879	102	1.00	0.74–1.34	0.76	0.34–1.72	0.96	0.73–1.28
32–40	10 113	270	1.00		1.00		1.00	
Professionals ^d^								
>48	6197	184	1.16	0.95–1.41	1.27	0.88–1.85	1.18	0.99–1.41
41–48	12 903	336	1.00	0.85–1.16	1.05	0.78–1.42	1.01	0.88–1.16
32–40	74 284	1968	1.00		1.00		1.00	
Technicians and associate professionals ^d^								
>48	5223	140	1.03	0.82–1.29	1.20	0.81–1.76	1.07	0.88–1.30
41–48	10 275	280	1.02	0.87–1.20	1.13	0.85–1.50	1.05	0.91–1.21
32–40	82 294	2473	1.00		1.00		1.00	
Workers in occupations that require basic level skills ^d^								
>48	8931	290	1.15	0.99–1.35	1.09	0.82–1.45	1.14	0.99–1.31
41–48	20 533	559	0.85	0.76–0.96	1.05	0.86–1.29	0.90	0.81–0.99
32–40	181 919	5695	1.00		1.00		1.00	
Workers in elementary occupations ^d^								
>48	2158	74	1.05	0.77–1.42	1.16	0.66–2.06	1.07	0.82–1.41
41–48	3128	106	1.03	0.79–1.33	1.02	0.59–1.76	1.02	0.81–1.30
32–40	37 807	1375	1.00		1.00		1.00	
Gainfully occupied people with unknown occupation ^d^								
>48	6476	193	0.99	0.82–1.21	0.95	0.63–1.44	0.99	0.82–1.18
41–48	6446	185	0.94	0.77–1.15	0.91	0.60–1.38	0.94	0.78–1.12
32–40	45 677	1499	1.00		1.00		1.00	
Workers aged 20–29 years ^e^								
>48	4797	111	1.03	0.80–1.32				
41–48	9477	190	0.86	0.70–1.04				
32–40	73 801	1787	1.00					
Workers aged 30–39 years ^e^								
>48	8844	241	1.06	0.89–1.26				
41–48	16 471	412	0.92	0.81–1.06				
32–40	119 076	3458	1.00					
Workers aged 40–49 years ^e^								
>48	10 361	326	1.09	0.94–1.26				
41–48	16 859	506	0.97	0.86–1.10				
32–40	122 670	3971	1.00					
Workers aged 50–59 years ^e^								
>48	8717	300	1.11	0.95–1.29				
41–48	14 357	460	0.96	0.84–1.09				
32–40	116 547	4064	1.00					

a Adjusted for sex, age, night/shift work, calendar time and socioeconomic status.

b Adjusted for age, night/shift work, calendar time and socioeconomic status.

c Adjusted for sex, age, calendar time and socioeconomic status.

d Adjusted for sex, age, night/shift work and calendar time.

e Adjusted for sex, night/shift work, calendar time and socioeconomic status.

**Table 2:** Rate ratio (RR) with 95% confidence interval (CI) for psychiatric hospital treatment due to mood, anxiety or stress-related disorders, as a function of weekly working hours among employees in Denmark in the calendar years 2000–2013.

Weekly working hours	Persons	Person years	Cases	RR ^a^	95% CI
>48	7993	38 628	78	0.96	0.76–1.21
41–48	13 592	66 186	132	0.90	0.75–1.08
32–40	109 736	531 859	1270	1.00	

a Adjusted for sex, age, night shift work, calendar time of the interview and socioeconomic status.

The results of a series of pre-specified sensitivity analyses are given in the supplementary material. None of the sensitivity analyses altered the statistical conclusions of the primary analysis. However, the analyses with stratification into industries may suggest increased risks of redeemed prescription for psychotropic drugs for >48 hours a week in the industrial groups of agriculture, forestry, hunting and fishing (estimated RR 1.49, 99% CI 0.95–2.36 ), construction (estimated RR 1.47, 99% CI 1.00–2.15) and human health and social work activities (estimated RR 1.27, 99% CI 1.02–1.58).

As part of the sensitivity analyses, we estimated the RR of relapse among employees with a past record of psychiatric treatment (Population 3, table S2), because we hypothesized that this group might be more vulnerable to long working hours than groups without past records of psychiatric treatment. To our knowledge, it is the first time ever that relapse rates have been estimated as a function of weekly working hours. The results did, however, not support the hypothesis of increased risk of working long hours within this group. With 32–40 working hours per week as reference, the estimated relapse RR were 0.94 (99% CI 0.79–1.13) for 41–48 working hours and 1.03 (99% CI 0.82–1.29) for >48 working hours.

## Discussion

In the present study, we found a statistically significant association between weekly working hours and redeemed prescriptions for psychotropic drugs with a U-shaped rate ratio pattern. The U-shaped pattern was present in all of the examined gender and age group strata, but not in all SES-groups or among night shift workers. It may, however be difficult to detect U-shaped relationships in small groups due to random errors. The U-shaped pattern was furthermore present in the analysis of psychiatric hospital treatment for mood, anxiety or stress-related disease.

Statistically significant U-shaped relationships between weekly working hours and adverse health outcomes have previously been observed or estimated for cardiovascular disease in Korea (8), cardiovascular mortality in Italy (9) and all-cause mortality in Denmark (10). A U-shaped relationship between weekly working hours and mental ill health makes sense. On one hand, prolonged workweeks may increase family incomes, which are known to be inversely associated with the risk of developing mental health problems (11–13). On the other hand, it is known that excessively long working hours may lead to short sleep and fatigue due to insufficient recovery between work shifts (14–19), which have been associated with an increased risk of developing mental health problems (20–26). From this viewpoint, we may expect the risk of mental ill health to decrease with weekly working hours up to a point where they become too long to allow sufficient sleep and recovery. Another explanation may be that employees in the reference group (32–40 hours per week) who work less than the standard 37 hours per week in Denmark, do so for a reason, which could in some cases be a lower threshold of stress, depression or anxiety.

In the present study, we tested the null hypothesis, which states that the expected RR for incident use of psychotropic drugs in the three exposure categories are equal versus the alternative hypothesis that they are not. The null hypothesis was rejected (P=0.003). The relationship between the estimated RR with 32–40 hours a week as reference was U-shaped, and the RR between the exposure categories >48 versus 41–48 hours a week was estimated at 1.15 (99% CI 1.03–1.28). Since the alternative hypothesis did not specify any particular shape of the relationship between weekly working hours and the examined rates (eg, monotonically increasing, monotonically decreasing, U-shaped), we may regard the test as an exploratory hypothesis test. To back up the U-shaped association, it should ideally be statistically tested in an independent data set with an a priori research hypothesis that explicitly states that the expected pattern is U-shaped.

It has previously been suggested that the association between long working hours and mental ill health depends on age, gender and SES (27). It has, moreover, been hypothesized that the effect of long working hours on the risk of mental ill health is stronger among employees with shift work than among employees without shift work (4). We could not confirm any of these interaction hypotheses; neither in the present study alone nor when data were pooled with our previous study.

In the present study, we used two different proxy measures for mental ill health. The RR for the contrast >48 versus 32–40 hours a week were estimated at 1.08 for redeemed prescription of psychotropic drugs and at 0.96 for psychiatric hospital treatment due to mood, anxiety or stress-related disease. These RR align quite well with the results obtained among western samples in the meta-analysis by Virtanen et al, where the pooled RR for development of depressive symptoms among workers with >54 versus 35–40 hours a week was estimated to be 1.11 in Europe, 0.97 in North America and 0.95 in Australia. The estimated RR of the present study are, however, substantially lower than the pooled risk ratio for development of depressive symptoms among long versus standard working hours in Asian countries, which Virtanen et al estimated to be 1.50. The difference between the Asian and Danish RR may due to the low prevalence of workers with very long hours in Denmark. Only 6.5% of the study population worked >48 hours a week. Approximately half of these worked <55 hours a week and only 20% of them worked >60 hours a week (cf. supplementary table S6).

We wanted the clinical endpoint of the psychotropic drug analysis to be the same as the one used in our previous study (12) (the study that we aimed to replicate). Thus, we included the entire group N05, which contains the subcategories N05A (antipsychotics), N05B (anxiolytics) and N05C (hypnotics and sedatives). We also included the entire group N06, which covers N06A (antidepressants), N06B (psychostimulants), N06C (antidepressants in combination with psycholeptics) and N06D (antidementia drugs).The relevance of psychostimulants and antidementia drugs in the study of associations between long working hours and mental ill health may be questionable. However, in our previous study, only 0.4% of the cases were due to psychostimulants and there were no cases of antidementia drugs, which indicates that it is unlikely that the inclusion of these two types of drugs have altered any statistical conclusions. The reason for including antipsychotics is that they often are used to treat depressive disorders (28).

It is possible that some types of mental disorders are associated with long working hours while others are not. It is, moreover, possible that an association between long working hours and mental disorders will be missed if the case definition includes too many diagnoses that have nothing to do with weekly working hours. To decrease the risk of missing an association due to dilution, we excluded psychiatric diagnoses that we, a priori, deemed unlikely to have long weekly working hours in their causal path. We included manic and bipolar disorders (F30, F31) in the case definition, and this choice may be questioned. The numbers of cases in these categories were, however, too low to affect the results of the study in any serious way.

The sensitivity analyses suggested a slightly elevated risk among employees with a stable exposure to >48 working hours per week (table S1). They also suggested that the association between weekly working hours and development of mental ill health might depend on industrial sector (table S4). In particular, they suggested a weak positive association between weekly working hours and incident use of psychotropic drugs in the following industrial groups: agriculture, forestry, hunting and fishing; construction; and human health and social work activities. It is possible that the high RR in these industries were due to high prevalences of work-related musculoskeletal injuries and disorders (29–32). It has been shown that musculoskeletal pain is an important predictor of depression, and it has been estimated that a third of the observed cases of redeemed prescriptions for antidepressants or psychiatric hospital treatment due to depression in the Danish labor force could be attributed to musculoskeletal pain (33). It is, moreover, reasonable to believe that the risk of incurring a work-related musculoskeletal injury or disorder depends on how many hours one spends at work. Another possible explanation for the high RR is that they were due to chance. In the present study, we estimated and reported a total of 91 RR and CI and it is therefore not surprising that some of the CI do not contain unity.

We followed the participants in national registers, which cover the entire target population, and thereby eliminated bias from missing follow-up data (except for ten persons not identified in registers). We were able to investigate effects of former cases of psychiatric treatment and could thereby rule out bias from pre-existing mental health problems (table S2). Another advantage of the present study is that the number of participants was large enough to allow us to: (i) differentiate between overtime work within and beyond the limit (48 working hours a week) of the EU Working Time Directive (5), (ii) supplement the analysis of psychotropic drug usage with estimated RR for psychiatric hospital treatment due to mood, anxiety or stress-related disease, and (iii) supplement the analysis of incident use of psychotropic drugs with an analysis of relapse rates among employees with a past record of psychiatric treatment.

The analyses were governed by a study protocol (6) that was accepted for publication before we linked the exposure data of the project to its outcome data. The analyses were, however, not completely blinded since the exposure data of the project have previously been analyzed in relation to ischemic heart disease (34), stroke (35), injuries (36) and all-cause mortality (10). There were two protocol violations. Firstly, the participants who were interviewed in 2013 were followed for a maximum period of four instead of the stipulated five years. The reason for this violation is that the project only had access to hospital treatment data up to 31 December 2017, which we had overlooked when we wrote protocol. The first violation is negligible since it only decreased the total number of person years at risk with approximately 1.4%. Secondly, we did not abide by the following protocol stipulation: “We will moreover exclude all participants who were registered in the employment classification module (ECM) as unemployed or otherwise not economically active during the main part of the calendar year preceding the start of the follow-up.” The purpose of this stipulation was to delete participants with unstable employment status, and the reason for the violation is that our project only had access to employment status according to the DLFS but not according to the ECM, which we had overlooked when we wrote the protocol. Our sensitivity analysis where we only included participants with stable employment status and stable weekly working hours (table S1) suggest, however, that the effect of the second violation was small.

The major drawback of the present study is that it is observational. Since it is not a randomized controlled trial, we cannot rule out the possibility that the results have been influenced by detection bias, referral bias, prescription bias and bias due to self-selection into working hour categories. The response rates have declined during the time of inclusion to 53%. It cannot be ruled out that employees with subclinical or emerging mental health problems could have a lower response rate than others, which may cause an underestimation of the true effect.

### Concluding remarks

To allow for possible effects of selection bias, misclassifications and uncontrolled confounding, Monson’s guide to strength of association recommends that a RR obtained in an observational cohort study should be interpreted as “no association” (too weak to be detected by epidemiologic methods) if it lies within the CI 0.9–1.2 (32). Although the main test for an association between weekly working hours and redeemed prescriptions for psychotropic drugs was statistically significant in the present study, we note that all RR that were estimated in the primary analyses of the study (tables 1 and 2) lie within Monson’s “no association” region. We also note that all of the RR obtained after pooling with our previous study lie within this region. Moreover, not only the RR but also the 99% CI of the pooled non-stratified analyses lie within the no association region. We may therefore safely conclude that, in this large prospective study, we found that long working hours are not an important predictor of mental ill health in the general working population of Denmark where long hours are less prevalent and extensive than in some of the countries reporting stronger associations.

## Supplementary material

Supplementary material
