# Supplementary material for: Long working hours and psychiatric treatment: A Danish follow-up study
Source: Scand J Work Environ Health. 2021 Mar 31;47(3):191–9. doi: 10.5271/sjweh.3936 (PMC8126440; doi:10.5271/sjweh.3936)
Supplement: Supplementary material [file SJWEH-47-191-S001.pdf]

Long working hours and psychiatric treatment: A Danish follow-up study[1]

by Harald Hannerz, FilLic,[2] Karen Albertsen, PhD, Martin Lindhardt Nielsen, PhD, Anne Helene Garde, PhD

1. *Supplementary material*
2. *Correspondence to: Harald Hannerz, National Research Centre for the Working Environment, Lersø Parkallé 105, DK-2100 Copenhagen, Denmark. [E-mail: hha@nfa.dk].*

Results of a series of protocol-based sensitivity analyses

In our study protocol [Hannerz et al., 2020], we defined a series of sensitivity analyses of associations between working time arrangements and use of psychotropic drugs among employees in the Danish labor force. The present supplementary appendix contains the results of the sensitivity analyses that concerns effects of weekly working hours. Each of the result tables is introduced with a short description of the purpose and method of the sensitivity analysis in question. The text of the descriptions has been adapted (copied and/or slightly amended) from the corresponding text of our study protocol [Hannerz et al., 2020], which contains the following copyright and license information:

"©Harald Hannerz, Karen Albertsen, Martin Lindhardt Nielsen, Anne Helene Garde. Originally published in JMIR Research Protocols (http://www.researchprotocols.org), 07.06.2020. This is an open-access article distributed under the terms of the Creative Commons Attribution License (https://creativecommons.org/licenses/by/4.0/), which permits unrestricted use, distribution, and reproduction in any medium, provided the original work, first published in JMIR Research Protocols, is properly cited. The complete bibliographic information, a link to the original publication on http://www.researchprotocols.org, as well as this copyright and license information must be included."

Stable exposure to weekly working hours

To find out if the estimated strength of the association between weekly working hours and redeemed prescriptions for psychotropic drugs increases when exposure is more stable over time, we conducted a sensitivity analysis, which only included people who i) participated in more than one interview, ii) were between 20 and 59 years old during their last interview, iii) were employed 32 or more working hours a week according to their first as well as their last interview, and iv) did not move more than one step among the ordered working time categories between the first and last interview. The included participants were thereafter categorized into 32 – 40; 41 – 48; > 48 hours a week, according to the mean of the reported usual working hours during their first and last interview. The follow-up of the included participants commenced at the very end of the calendar year of their last interview. The statistical model was otherwise the same as in the primary analysis. The results of this sensitivity analysis are given in Table S1.

Table S1. Rate ratio (RR) with 99% confidence interval (CI) for incident use of psychotropic drugs, as a function of weekly working hours among employees in Denmark in the calendar years 2004 – 2013

Weekly working hours	Persons	Person years	Cases	RR*	99% CI
> 48	3215	12 808	386	1.16	1.01 - 1.33
41 – 48	10 194	40 843	1092	0.97	0.89 - 1.06
32 – 40	75 654	271 728	7955	1.00	-

* Adjusted for sex, age, night shift work, calendar time of the interview and socioeconomic status

Controlling for possible bias due to preexisting mental health problems

In the primary analysis, we excluded participants who received psychiatric hospital treatment or redeemed a prescription for psychotropic drugs during the calendar year preceding the start of the follow-up period. It was, however, possible that the results of the primary analysis would be influenced by cases that occurred earlier than one year prior to baseline. To explore this possibility, we conducted a sensitivity analysis in which the sample was stratified into two cohorts. The first cohort excludes all participants who received psychiatric hospital treatment or redeemed a prescription for psychotropic drugs some time during a five-year period prior to the start of follow-up. The second cohort consists of the participants who were excluded from the first cohort due to psychiatric hospital treatment or redeemed prescription for psychotropic drugs within year 2-5 prior to the start of follow up. This particular analysis included only participants who lived in Denmark throughout the concerned five-year period. Moreover, it included only people who participated in Danish Labor Force Survey sometime during the calendar period 2004 – 2013. The statistical methods and inclusion criteria of the analysis were otherwise the same as in the primary analysis. The results are shown in Table S2. The results of the first cohort (population 2) are interpreted as incidence rate ratios while the results of the second cohort (population 3) are interpreted as relapse rate ratios.

Table S2. Rate ratio with 99% confidence interval (CI) for incident or recurrent use of psychotropic drugs, as a function of weekly working hours among employees in Denmark 2004 - 2013

Type of population*	Weekly Working hours	Person years	Cases	RR**	99% CI
1. Workers with no occurrences*** during the first year prior to baseline.	> 48	20 226	588	1.09	0.97 - 1.22
	41 - 48	34 063	901	0.94	0.86 - 1.03
	32 - 40	295 849	8660	1.00	-
2. Workers with no occurrences during the first to fifth year prior to baseline.	> 48	18 832	444	1.10	0.97 - 1.26
	41 - 48	31 755	679	0.95	0.86 - 1.06

	32 - 40	273 741	6368	1.00	-
3. Workers with no occurrences during the first year prior to baseline, but at least one occurrence during the second to fifth year prior to baseline.	> 48	1393	144	1.03	0.82 - 1.29
	41 - 48	2308	222	0.94	0.79 - 1.13
	32 - 40	22 108	2292	1.00	-

* Population 1 excludes former cases by use of the same principle that was used in the primary analysis. I.e. workers who were treated in the one-year period preceding baseline are excluded. Population 2 and 3 are disjoint and exhaustive subsets of population 1.

** Adjusted for sex, age, night shift work, calendar time of the interview and socioeconomic status

*** With “occurrences” we mean, “occurrences of redeemed prescriptions for psychotropic medicine or psychiatric hospital treatment”.

Estimated rate ratios without exclusion of prevalent cases

The estimated rate ratios of redeemed prescriptions for psychotropic drugs as a function of weekly working hours, without exclusion of prevalent cases are given in table S3.

Table S3. Rate ratio (RR) with 99% confidence interval (CI) for incident use of psychotropic drugs, as a function of weekly working hours among employees in Denmark in the calendar years 2000 – 2013, without exclusion of prevalent cases

Weekly working hours	Persons	Person years	Cases	RR*	99% CI
> 48	8614	33 596	1460	1.03	0.96 - 1.11
41 – 48	14 577	58 515	2337	0.90	0.85 - 0.95
32 – 40	119 664	444 329	21 203	1.00	-

* Adjusted for sex, age, night shift work, calendar time of the interview and socioeconomic status

Controlling for industrial sector

In order to pool results of the present study with results obtained in our previous study [Hannerz and Albertsen, 2016] we used the same covariates in the primary analysis of the present study as we did in our previous study. The primary analysis therefore controls for an occupational-based SES, but it does not control for industrial sector, which has been shown to be a predictor for mood disorders in the general working population of Denmark [Hannerz et al., 2009]. We wanted to know if the results of the present study would change if we added industrial sector to the model and therefore conducted a sensitivity analysis where we firstly controlled for (Table S4) and thereafter stratified by (Table S4) industrial sector. The statistical methods and inclusion criteria of the analysis were otherwise the same as in the primary analysis.

Table S4. Rate ratio (RR) with 99% confidence interval (CI) for incident use of psychotropic drugs, as a function of weekly working hours among employees in Denmark in the calendar years 2000 – 2013

Weekly working hours	Persons	Person years	Cases	RR*	99% CI
> 48	7993	32 718	978	1.08	0.99 - 1.18
41 – 48	13 592	57 164	1568	0.93	0.87 - 1.00
32 – 40	109 736	432 094	13 280	1.00	-

* Adjusted for sex, age, night shift work, calendar time of the interview, socioeconomic status and industry

Table S5. Industry specific rate ratio (RR) with 99% confidence interval (CI) for incident use of psychotropic drugs, as a function of weekly working hours among employees in Denmark in the calendar years 2000 – 2013

Industry	Weekly Working hours	Persons	Person years	Cases	RR*	99% CI
Agriculture, forestry, hunting and fishing	> 48	321	1297	41	1.49	0.95 - 2.36
	41 - 48	273	1130	26	1.13	0.65 - 1.95
	32 - 40	1518	6228	146	1.00	-
Manufacturing, mining and quarrying	> 48	1068	4472	115	0.97	0.76 - 1.25
	41 - 48	1931	8380	219	0.97	0.81 - 1.16
	32 - 40	19 781	80 600	2403	1.00	-
Construction	> 48	336	1388	48	1.47	1.00 - 2.15
	41 - 48	685	3010	68	0.97	0.70 - 1.34
	32 - 40	7808	31 902	756	1.00	-
Wholesale and retail trade; repair of motor vehicles	> 48	1055	4457	113	1.03	0.80 - 1.33
	41 - 48	1899	8163	196	0.95	0.78 - 1.16
	32 - 40	13 989	55 114	1503	1.00	-
Transporting and storage	> 48	1073	4515	131	1.03	0.81 - 1.32
	41 - 48	914	3988	94	0.82	0.62 - 1.09
	32 - 40	5755	23 073	694	1.00	-
Accommodation and food service activities	> 48	156	633	15	0.77	0.38 - 1.52
	41 - 48	209	865	30	1.07	0.65 - 1.77
	32 - 40	1682	6357	213	1.00	-
Human health and social work activities	> 48	839	3157	147	1.27	1.02 - 1.58
	41 - 48	2561	10 380	365	0.86	0.75 - 1.00

	32 - 40	19 085	73 535	2822	1.00	-
Other	> 48	2965	12 177	349	1.07	0.93 - 1.24
	41 - 48	4982	20 740	557	0.99	0.88 - 1.11
	32 - 40	38 690	149 994	4455	1.00	-
Missing	> 48	180	623	19	0.61	0.33 - 1.13
	41 - 48	138	508	13	0.51	0.25 - 1.07
	32 - 40	1428	5291	288	1.00	-

* Adjusted for sex, age, night shift work, calendar time of the interview and socioeconomic status

Post hoc estimation of rate ratios with an alternative categorization of weekly working hours

Table S6. Post hoc estimated rate ratio (RR) with 99% confidence interval (CI) for incident use of psychotropic drugs, as a function of weekly working hours among employees in Denmark in the calendar years 2000 – 2013

Weekly working hours	Persons	Person years	Cases	RR*	99% CI
> 67	1074	4184	137	1.12	0.89 – 1.40
61 - 67	565	2289	67	1.05	0.76 – 1.44
55 - 60	2321	9464	278	1.08	0.93 – 1.27
49 - 54	4033	16 780	496	1.08	0.96 – 1.22
41 - 48	13 592	57 164	1568	0.95	0.88 – 1.01
35 - 40	100 845	397 642	11 918	1.00	-
32 - 34	8891	34 451	1362	1.11	1.03 – 1.19

* Adjusted for sex, age, night shift work, calendar time of the interview and socioeconomic status

Post hoc estimation of rate ratios for depressive disorders

The following table was produced in response to a comment by a referee:

Table S7. Post hoc sensitivity analysis. Rate ratio (RR) with 95% confidence interval (CI) for psychiatric hospital treatment due to depressive mood disorder (ICD-10: F32-F33), as a function of weekly working hours among employees in Denmark in the calendar years 2000 – 2013

Weekly working hours	Persons	Person years	Cases	RR*	95% CI	99% CI
> 48	7993	38 717	43	1.32	0.96 - 1.81	0.86 – 2.01
41 – 48	13 592	66 381	58	0.96	0.73 – 1.26	0.67 – 1.37
32 – 40	109 736	533 568	524	1.00	-	-

* Adjusted for sex, age, night shift work, calendar time of the interview and socioeconomic status

References

1. Hannerz H, Albertsen K. Long working hours and use of psychotropic medicine: a follow-up study with register linkage. Scand J Work Environ Health. 2016 Mar;42(2):153-61. doi: 10.5271/sjweh.3550.
2. Hannerz H, Albertsen K, Nielsen ML, Garde AH. Prospective associations between working time arrangements and psychiatric treatment in Denmark: a study protocol. JMIR Res Protoc. 2020.
3. Hannerz H, Tüchsen F, Pedersen BH, Dyreborg J, Rugulies R, Albertsen K. Work-relatedness of mood disorders in Denmark. Scand J Work Environ Health. 2009;35(4):294-300.
